# The *A2B trial*, antibiotic prophylaxis for excision-graft surgery in burn patients: a multicenter randomized double-blind study

**DOI:** 10.1186/s13063-020-04894-y

**Published:** 2020-11-25

**Authors:** François Dépret, Boris Farny, Mathieu Jeanne, Kada Klouche, Thomas Leclerc, Karine Nouette-Gaulain, Olivier Pantet, Francis Rémerand, Antoine Roquilly, Anne-Françoise Rousseau, Simon Sztajnic, Sandrine Wiramus, Eric Vicaut, Matthieu Legrand, for the A2B trial investigators, François Dépret, Matthieu Legrand, Thomas Leclerc, Boris Farny, Sandrine Wiramus, Antoine Roquilly, Mathieu Jeanne, Francis Rémerand, Kada Klouche, Simon Sztajnic, Karine Nouette, Barraud Damien, Anne-Françoise Rousseau, Olivier Pantet

**Affiliations:** 1 Department of Anaesthesiology, Critical Care Medicine and Burn unit, AP-HP, Saint Louis and Lariboisière University Hospitals, 1 Avenue Claude Vellefaux, 75010 Paris, France; 2 INSERM UMR-S942, Institut National de la Santé et de la Recherche Médicale (INSERM), FHU PROMICE Lariboisière Hospital, Paris, France; 3 INI-CRCT Network, Nancy, France; 4 University of Paris, F-75475 Paris, France; 5 Department of Anesthesiology, Critical Care and Burn Unit, Saint-Joseph Saint-Luc Hospital, Lyon University, Lyon, France; 6 CHU Lille, Anaesthesia and Critical Care, Burn Centre, 59000 Lille, France; 7 University of Lille, Inserm, CHU Lille, CIC 1403, 59000 Lille, France; 8 University of Lille, EA 7365 - GRITA, 59000 Lille, France; 9 Department of Intensive Care Unit, Hospital Lapeyronie, 55045 Montpellier, France; 10 Percy Military Teaching Hospital, Clamart, France; 11 Val-de-Grâce Military Medical Academy, Paris, France; 12 CHU Bordeaux, Service d’Anesthésie Réanimation Pellegrin, Hôpital Pellegrin, Place Amélie Raba Léon, F-33000 Bordeaux, France; 13 CHU Bordeaux, Department of Anaesthesia and Critical Care, Magellan Medico-Surgical Center, F-33000 Bordeaux, France; 14 Univ. Bordeaux, INSERM U12-11, Laboratoire de Maladies Rares: Génétique et Métabolisme (MRGM), 176 Rue Léo Saignat, F-33000 Bordeaux, France; 15 Medical Intensive Care, CHU Vaudois, Lausanne, Switzerland; 16 Département d’Anesthésie-Réanimation Chirurgicale, Centre Hospitalier Universitaire de Tours, Tours, France; 17 Department of Anaesthesia and Critical Care, Hôtel-Dieu, University Hospital of Nantes, Nantes, France; 18 Laboratoire UPRES EA 3826 “Thérapeutiques cliniques et expérimentales des infections”, University of Nantes, Nantes, France; 19 Department of Anesthesiology and Intensive Care, CHU Liège, Liège, Belgium; 20 Intensive Care Unit, Department of Anesthesia and Critical Care, University Hospital of Toulouse, Toulouse, France; 21 Department of Anaesthesia and Intensive Care Medicine and Burn Centre, University Hospital of Marseille, La Timone Hospital, Marseille, France; 22 APHP, Department of Biostatistics, Université Paris-Diderot, Sorbonne-Paris Cité, Fernand Widal Hospital, Paris, France; 23 Department of Anesthesia and Perioperative Care, University of California, 500 Parnassus Avenue MUE416, Box 0648, San Francisco, CA 94143 USA

**Keywords:** Burn, Antibiotic prophylaxis, Excision-graft

## Abstract

**Background:**

The indication for antibiotic prophylaxis in burn patients remains highly controversial, with no consensus having been reached. The objective of antibiotic prophylaxis is to reduce the risk of postoperative local and systemic infections. Burn surgery is associated with a high incidence of bacteremia, postoperative infections, and sepsis. However, antibiotic prophylaxis exposes patients to the risk of selecting drug-resistant pathogens as well as to the adverse effects of antibiotics (i.e., *Clostridium difficile* colitis). The lack of data precludes any strong international recommendations regarding perioperative prophylaxis using systemic antibiotics in this setting.

The goal of this project is therefore to determine whether perioperative systemic antibiotic prophylaxis can reduce the incidence of postoperative infections in burn patients.

**Methods:**

The A2B trial is a multicenter (10 centers), prospective, randomized, double-blinded, placebo-controlled study. The trial will involve the recruitment of 506 adult burn patients with a total body surface area (TBSA) burn of between 5 and 40% and requiring at least one excision-graft surgery for deep burn injury. Participants will be randomized to receive antibiotic prophylaxis (antibiotic prophylaxis group) or a placebo (control group) 30 min before the incision of the first two surgeries. The primary outcome will be the occurrence of postoperative infections defined as postoperative sepsis and/or surgical site infection and/or graft lysis requiring a new graft within 7 days after surgery. Secondary outcomes will include mortality at day 90 postrandomization, skin graft lysis requiring a new graft procedure, postoperative bacteremia (within 48 h of surgery), postoperative sepsis, postoperative surgical site infection, number of hospitalizations until complete healing (> 95% TBSA), number of hospitalization days living without antibiotic therapy at day 28 and day 90, and multiresistant bacterial colonization or infection at day 28 and day 90.

**Discussion:**

The trial aims to provide evidence on the efficacy and safety of antibiotic prophylaxis for excision-graft surgery in burn patients.

**Trial registration:**

ClinicalTrials.gov NCT04292054. Registered on 2 March 2020

**Supplementary Information:**

The online version contains supplementary material available at 10.1186/s13063-020-04894-y.

## Background

The indication for antibiotic prophylaxis in burn patients remains highly controversial, with no consensus having been reached. The objective of antibiotic prophylaxis is to reduce the risk of postoperative local and systemic infections. Burn surgery is associated with a high risk of bacteremia, postoperative infections, and sepsis [1–6]. Furthermore, infections and sepsis represent frequent complications in burn patients, being the main cause of death or prolonged hospitalization [7]. However, antibiotic prophylaxis exposes patients to the risk of selecting drug-resistant pathogens as well as to the adverse drug reactions of antibiotics (i.e., allergies, *Clostridium difficile* colitis) [8, 9]. Due to prolonged intensive care unit (ICU) stays and immunosuppression, burn patients are highly exposed to the risk of multidrug-resistant bacterial infections, and the emergence of bacteria with antibiotic resistance represents a major threat in this population.

The question of systemic antibiotic prophylaxis is a major unresolved issue in the management of burn patients, with no existing recommendations with strong evidence. Existing data include small studies at unclear or high risk of bias [10, 11]. To date, no randomized study of sufficient methodological quality has addressed this question [12]. Furthermore, recommendations regarding perioperative prophylaxis using systemic antibiotics vary across sources (with some limiting perioperative prophylaxis to only those with severe burns > 40% total body surface area) [13]. The lack of data precludes any strong international recommendations regarding an antibiotic prophylaxis strategy.

The aim of this study (named the A2B trial) was therefore to determine whether preoperative antibiotic prophylaxis before excision/graft surgeries can reduce the incidence of postoperative infections and/or autograft lysis in burn patients.

The results of this study will provide important high-quality data to guide physicians treating burn patients and impact future guidelines.

## Methods/design

### Aim, design, and setting of the study

**Fig. 1:**
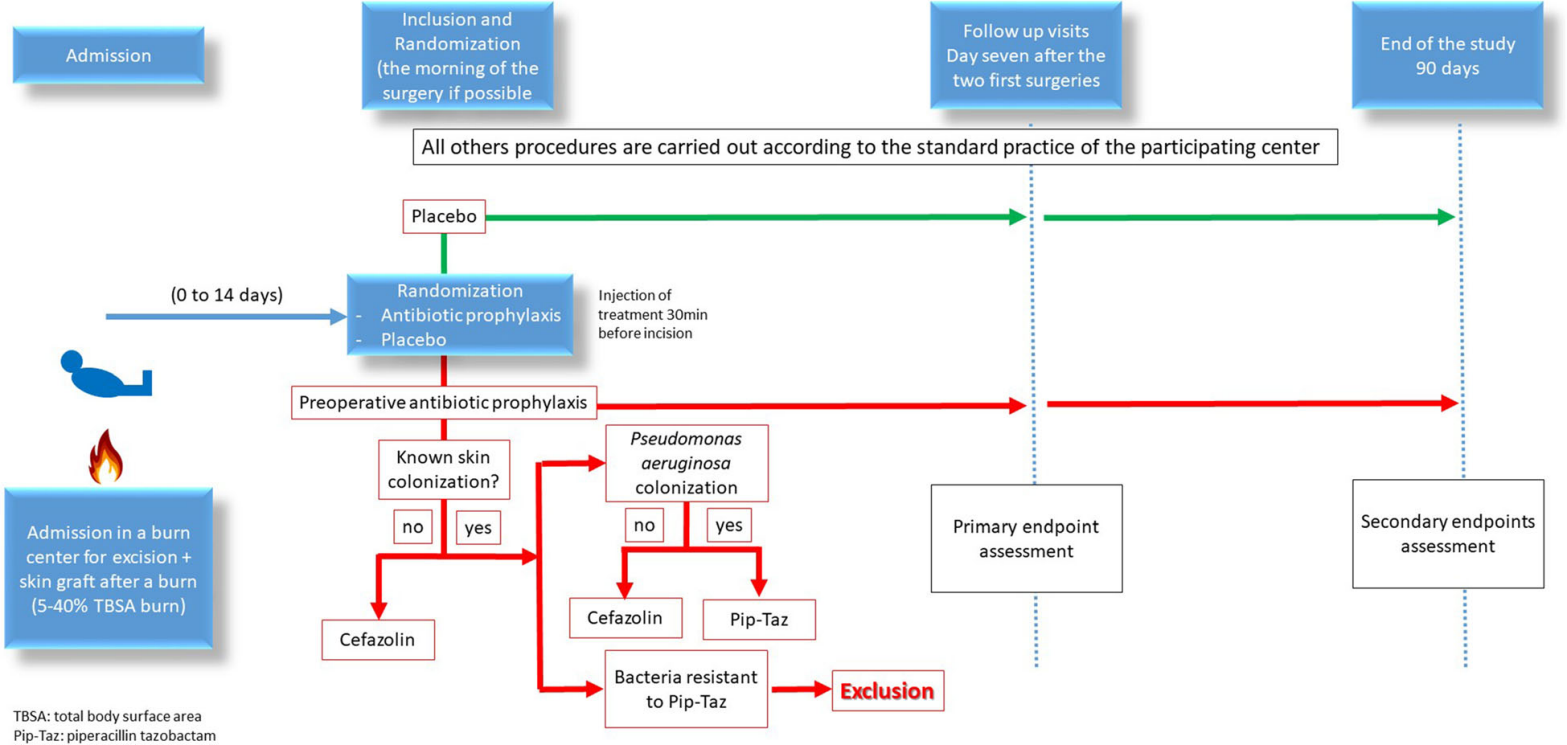
Study design. TBSA, total body surface area; Pip-Taz, piperacillin-tazobactam

The study protocol was designed in accordance with the SPIRIT guidelines [14] (Fig. 1). The A2B trial is an academic, investigator-initiated multicenter (10 centers), prospective, parallel-group (two groups), double-blinded, placebo-controlled, randomized, trial of antibiotic prophylaxis administration to adults with deep burns admitted to a participating burn intensive care unit (BICU). The aim of this trial is to explore the impact of systemic antibiotic prophylaxis on postoperative infections (defined as sepsis, surgical site infections, or graft lysis) in burn patients with a total body surface area (TBSA) burn of between 5 and 40%. Exploratory secondary outcomes and adjusted analyses will also be conducted. A total of 506 patients with a TBSA between 5 and 40% requiring at least one excision-graft surgery for burn injury will be enrolled in 10 centers with experience in the management of severely burn patients (list of participating centers, Appendix 1).

The primary outcome is a composite outcome of postoperative infection defined as follows:

1. Postoperative sepsis. Postoperative sepsis is defined by the Sepsis-3 definition [15] within 7 days after surgery (Appendix 2)
2. And/or surgical site infection (Appendix 2) requiring treatment with at least 5 days of systemic antibiotic therapy
3. And/or graft lysis (diagnosed within 7 days after surgery) needing a new graft procedure (Appendix 2)

The secondary outcomes are as follows:

- Mortality at day 90
- Skin graft lysis (diagnosed within 7 days after surgery) requiring a new graft procedure
- Surgical site infection requiring treatment with at least 5 days of systemic antibiotic therapy (diagnosed within 7 days after surgery)
- Postoperative bacteremia (within 48 h of surgery)
- Postoperative sepsis (diagnosed within 7 days after surgery)
- Number of days of hospitalization until complete healing (> 95% total burn surface area)
- Number of hospitalization days alive without antibiotic therapy at day 28 and day 90
- And multidrug-resistant bacterial colonization or infection at day 28 and day 90

### Characteristics of participants

All participants will be included and randomized by the clinician in charge of the patient. To be included, patients must meet all the inclusion criteria:

1. Patients over 18 years of age with signed informed consent or inclusion under the emergency provisions of the law (Article L1122-1-3 of the public health code [PHC]/modified by order no. 2016-800 of June 16, 2016—art. 2)
2. Patients with a TBSA between 5 and 40% requiring at least one excision-graft surgery for burn injury
3. Signed informed consent or inclusion under the emergency provisions of the law (article L1122-1-2 of the PHC)

Patients are excluded if any of the following criteria apply:

1. Proven severe allergy to cephalosporin or piperacillin-tazobactam or any other antibacterial agent of the penicillin class
2. History of severe allergic reaction to any other beta-lactam (e.g., cephalosporins, monobactams, or carbapenems)
3. Patient on antibiotic therapy at the time of inclusion
4. Pregnant or breast-feeding patient
5. Patient transferred from another burn unit
6. Patient participants in investigational competitive medicinal product studies on the primary endpoint
7. Patients with local or systemic signs of infection requiring immediate systemic antimicrobial therapy
8. Patient under guardianship
9. Patient under curatorship
10. Patient not covered by the social security
11. Known colonization of the burned area to be excised with tazocillin-resistant germ
12. Obesity

### Randomization process

Patients are allocated to treatment with antibiotic prophylaxis or a placebo at a ratio of 1:1. A randomization number will be assigned during randomization. This number will have the following format: R-XXXX (R+4 numerical positions). The randomization list will be performed electronically through the CleanWeb application at the clinical research unit “Lariboisière-St Louis” and stratified by center and according to the percentage of burn total body surface area (TBSA 5–20% and 21–40%). The randomization list will be developed by a different biostatistician than the biostatistician who will conduct the final analysis within the CRU “Lariboisière- St Louis.”

The randomization will be performed after written consent obtainment and as late as possible before surgery by web software (CleanWeb), which assigns the patient a randomization number.

Information about the person who volunteers to a research, the specificity for obtaining consent of the person who is part of the research, and the informed consent document are provided in supplementary material file 1.

### Bacterial skin colonization

Bacterial colonization of *Pseudomonas aeruginosa* will be detected using wound swabs before surgery. If no swabs can be performed before the surgery and colonization is unknown, the patient will be considered not colonized by *Pseudomonas aeruginosa*.

### Drug being tested

After randomization, a prescription indicating the group corresponding to the arm in which the patient has been assigned will be edited automatically (through CTMS/CleanWeb) and printed out.

The antibiotic or placebo will be injected 30 min before starting the surgical procedure. The median number of surgical procedures in these patients is 1 (95% CI 1–2) (unpublished personal data). In the case of multiple surgical procedures, the same protocol will be applied to the second procedure (i.e., placebo infusion in the control group, antibiotic prophylaxis in the interventional group) but not beyond. These cases are, however, expected to represent only a few cases. In the intervention group, antibiotic prophylaxis will be a cephalosporin (cefazolin 2 g over 30 min infusion) if the colonization is unknown or in the absence of colonization with *Pseudomonas aeruginosa* or piperacillin-tazobactam (4 g over 30 min infusion) if the burn wound is colonized with *Pseudomonas aeruginosa*.

The administration period will be followed by a clinical follow-up period of 90 days. Patients are closely monitored in the first 7 days after randomization (Fig. 1).

No treatment will be prohibited with respect to the research.

### Data collection

Data on all patients will be collected by trained study nurses or physicians using a web-based (e-CRF). Data collected and time points are presented in Table 1. Monitoring is performed by the clinical research organization and the sponsor.

**Table 1:** SPIRIT schematic schedule of enrollment, interventions, and assessments

	Inclusion	Before the surgical procedure(s)^**a**^	First 7 days after surgery surgical procedures	Day 28	Day 90 after first surgery
Informed consent (if not given at the previous visits) (according to law L1122-1-3 of the PHC/Order No. 2016–800 of June 16, 2016—art. 2)	X		X^(b)^		X^(b)^
Inclusion and non-inclusion criteria	X				
Randomization	X				
Medical history/comorbidities	X		X		X^d^
Antibiotic prophylaxis or placebo		X			
Postoperative infections			X		X^d^
Pictures of the burn area/surgical site		X	X		X^d^
Concomitant treatment	X	X	X		X^d^
Clinical^(c)^ examination/	X^(c)^		X^(c)^		X^d^
Skin bacterial colonization	X	X	X	X	X^d^
Glasgow coma score	X	X	X		X^d^
Assessment of SOFA score	X	X	X		
Retrieval of adverse events	X		X	X	X^d^
Beta HCG dosage	X	X			
Antibiotic prophylaxis or placebo		X			
Assessment of morbidity and mortality			X		X^d^
Assessment of primary endpoint		X			
Assessment of secondary endpoints			x	x	X^d^
Vital status				X^e^	X^e^

^a^The Beta HCG dosage will be performed before the first surgery, in the case of the second surgery if necessary and before the administration of the treatment (placebo or antibiotic)

^(b)^If not done at the previous visits (according to law L1122-1-2 of the PHC)

^(c)^Clinical examination:

- Hemodynamic parameters:

Systolic, mean and diastolic arterial pressure, heart rate

- Biological parameters:

Arterial plasma lactate level, plasma pH and base excess, PaO2 and PaO2/FiO2, PaCO2, blood urea nitrogen, serum creatinine, serum potassium level, hemoglobin, total bilirubin level, and platelet count

^d^If the patient is still hospitalized

^e^If the patient has been discharged from the hospital

Each component of the primary endpoint will be collected at each visit based on the previous definition (Appendix 2). The occurrence of postoperative infection will be collected by intensivists or infectious disease specialist consultants blinded to the interventional or control arm. Skin infection and skin graft lysis requiring a new graft procedure will be assessed by a surgeon blinded to the arm of the study at the respective center. Any serious adverse event will be notified by the investigator to the sponsor without delay. All data and other information generated will be held in strict confidence. The patients will be identifiable only by their initials and inclusion number. All documents that identify the patient (e.g., informed consent) are maintained in confidence by the investigator. The Standard Protocol Items: Recommendation for Interventional Trials (SPIRIT) reporting guidelines are applied [14] (SPIRIT checklist is provided as supplementary file 2). The results of the study will be communicated to the participants, healthcare professionals, and the public by publication and reporting in clinical trial databases (EudraCT, NCT) without restriction. Furthermore, the results of this study will be published in a peer-review medical journal with communication in an international scientific meeting.

### Quality control

Details about quality control, case report form, management of noncompliance, audit and inspection, blinding methods, and serious adverse event notification are specified in supplementary file 1.

### Statistical analysis

The primary aim of this trial is to demonstrate superiority in the intent-to-treat analysis of antibiotic prophylaxis infusion versus placebo on postoperative infection. The null hypothesis is that there are no differences in the postoperative infection rate between the two treatment groups.

The primary analysis is based on the analysis of the primary criterion in intention to treat.

The incidence of postoperative infection, adjusted according to %TBSA, will be compared between study arms using logistic regression with the percentage of burn total body surface area as a covariate. The efficacy of antibiotic prophylaxis will be considered to be proven if the null hypothesis for the primary endpoint is rejected and if the treatment difference is in favor of antibiotic prophylaxis in the sense of a shift to a lower postoperative infection rate under antibiotic prophylaxis.

Concerning secondary analysis, all parameters related to an event (i.e., 90-day mortality, skin raft lysis requiring a new graft procedure, postoperative bacteremia, pulmonary infection, surgical site infection, and multiresistant bacterial colonization) will be compared by logistic regression with the percentage of TBSA as a covariate. The time until complete healing will be described with Kaplan-Meier curves and analyzed using the Cox model. The reference time is defined as the randomization time. All patients will be censored at the time of the last observation.

The number of hospitalization days living without antibiotic therapy at day 28 and day 90 will be compared between groups by the Mann-Whitney test.

Continuous variables will be summarized using the number of observations, mean, standard deviation, minimum, maximum, 25%, 50%, 75% quartiles, and two-sided 95% confidence intervals. Means, medians, minimum, maximum, and standard deviations will be presented to one further decimal place.

Categorical variables will be expressed as absolute and relative frequencies (percentages).

Missing values will be imputed by a multiple imputation technique.

### Sample size determination

The incidence of the primary endpoint is estimated to be 25% (based on investigators’ personal data). With an alpha risk of 4.9% (adjusted for interim analysis) and power = 80%, the number of patients to include to show an absolute reduction of 10% of the primary endpoint, we need to enroll 506 patients (253 in the intervention group and 253 in the control group).

### Data monitoring and interim analysis

A steering committee will advise the conduct of the trial (Appendix 3). An independent Data and Safety Monitoring Committee (DSMC) was not required for this trial. An interim analysis will be performed after inclusion of 50% of the patients. It will allow termination of the study for efficacy, futility, or sample size reassessment (only an increase in *N* will be allowed for changes in sample size). The O-Brien-Fleming boundaries will be used for nominal alpha values (i.e., 0.0031 for interim analysis and 0.049 for final analysis).

The sponsor must notify all the investigators any information that could adversely affect the safety of the participants. Assistance Publique Hôpitaux de Paris (AP-HP) is the sponsor of this study and has delegated power to its Clinical Research and Development Department (DRCD) to conduct the study in accordance with Article L.1121–1 of the French Public Health Code. AP-HP reserves the right to terminate the study at any time for medical or administrative reasons. In this case, the investigator will be informed accordingly.

**Fig. 2:**
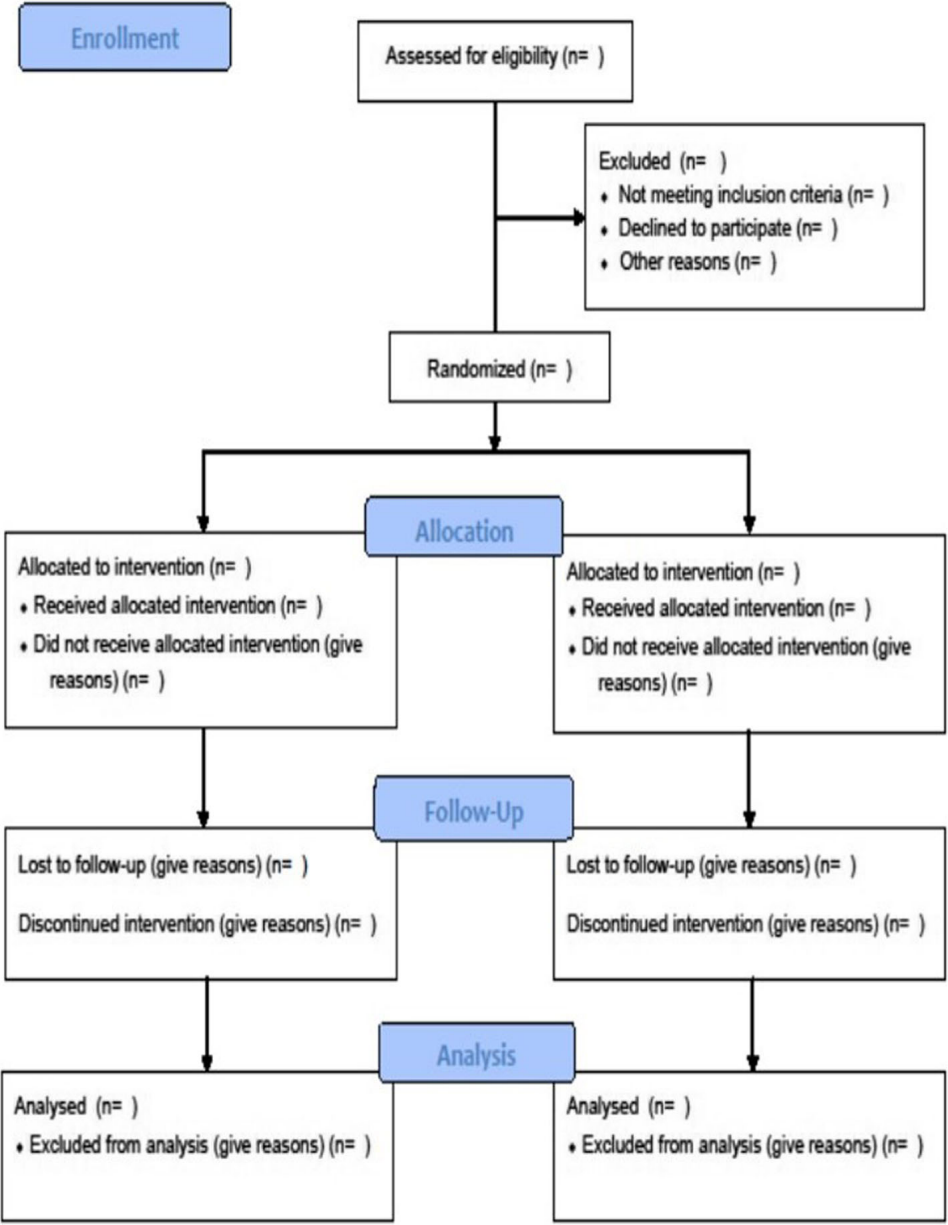
CONSORT flow chart of the study

In the case of loss to follow-up, the investigator will do his or her best to contact the patient to determine his/her vital status. Mailing address, phone number, and phone number of at least one relative will be collected at inclusion to contact the patient. If a patient leaves the research prematurely, data relating to the participant can be used unless an objection was recorded when the patient signed the consent form. If consent is withdrawn, no data about the participant may be used unless he/she states in writing that he/she does not object. In this case, the participant will be excluded from the research (Fig. 2).

It should be noted that all amendments will be validated by the institutional review board, and once validated, the content of the amendments will be communicated to all investigators through contact emails and newsletters (on a 3-month basis) and during the investigator meeting.

### Serious adverse event notification

According to Article R.1123-49 of the French PHC, the investigator must notify the sponsor without delay on the day when the investigator becomes aware of any serious adverse event that occurs during a trial as described in Article L.1121-1(1) PHC, except those which are listed in the protocol (see section 10.1.2.2.2) and, if applicable, in the investigator’s brochure as not requiring a notification without delay. These latter should be notified by the investigator to the sponsor in an appropriate delay taking into consideration the specific features of the trial, the serious adverse events, and the modalities specified in the protocol or the investigator’s brochure.

The sponsor will especially follow the serious adverse events listed below:

- Events with fatal outcome
- Anaphylactic shock
- Quincke edema

The investigator must notify the sponsor without delay on the day when the investigator becomes aware of emerging safety issues, as well as security measures taken.

The other events requiring the investigator to notify the sponsor without delay are as follows:

- Liver test abnormalities (ALT/AST three times higher than the upper limit)
- *Clostridium difficile* colitis
- Acute kidney injury

All these events will be reported in the trial publication.

## Discussion

The indication for antibiotic prophylaxis in burn patients remains highly controversial, with no consensus having been reached. To date, no strategy (antibiotic prophylaxis versus no antibiotic prophylaxis) has shown superiority [13]. The administration of antibiotic prophylaxis has the potential to improve clinical outcomes. The A2B trial aims to detect a beneficial effect of antibiotic prophylaxis on postoperative infections while minimizing any potential risk. Other important clinical outcomes, including survival, will be explored as secondary endpoints. The question of the impact of antibiotic prophylaxis on postoperative infections remains open in this selected population. In addition, the emergence of antibiotic-resistant bacteria is a major threat to this population. Framing the first two surgical procedures seems to be the most relevant way to evaluate the impact of antibiotic prophylaxis in burn patients.

Our protocol will be the most rigorous and the most likely to address the question of the impact of antibiotic prophylaxis on burn surgery postoperative infections. We expect that the antibiotic prophylaxis strategy will decrease the incidence of postoperative infections (i.e., postoperative sepsis, surgical site infection, and graft lysis). Antibiotic prophylaxis strategy is therefore expected to decrease the need for intensive care/hospital resources, allowing faster BICU/hospital discharge, thereby significantly decreasing costs and ultimately improving patient outcomes. Infection is the main cause of morbidity and mortality in burn patients [16].

We acknowledge that the impact of antibiotic prophylaxis in patients suffering very large burn injuries will need additional studies using an alternative design (e.g., Bayesian approach) due to the very low number of patients suffering very large burn injuries each year.

## Trial status

The current protocol is version 1.2, dated 19 December 2019. Recruitment will begin in September 2020. The approximate completion date for recruitment is in September 2024. This trial was prospectively registered before recruitment began. The study protocol was approved by the institutional review board (IRB) of Sud Est IV on 11 December 2019 (approval number 2019-002396-34), and from the agence nationale de sécurité du medicament et des produits de santé (MEDAECNAT-2019-10-00036), it was registered as a clinical trial on 2 March 2020 (NCT03788837) and in EudraCT (No. 2019-002396-34) (Supplementary files 3 and 4).

### Supplementary Information

**Additional file 1.** Details about quality control, case report form, management of noncompliance, audit and inspection, blinding methods, and serious adverse event notification

**Additional file 2.** SPIRIT checklist

**Additional file 3.** Institutional review board (IRB) of Sud Est IV approval

**Additional file 4.** Agence nationale de sécurité du medicament et des produits de santé approval

## Data Availability

Data sharing is not applicable to this article, as no datasets were generated or analyzed during the current study. However, trial data will be made available by the coordinating investigator upon reasonable request.

## Appendix 1

**Table 2:** A2B trial principal and deputy investigators

Name	First name	City	Country	Health facility
Dépret Legrand	François Matthieu	Paris	France	CHU Saint Louis
Leclerc	Thomas	Clamart	France	CH Percy
Farny	Boris	Lyon	France	CHU Edouard Herriot
Wiramus	Sandrine	Marseille	France	Hôpital de la Conception APHM
Roquilly	Antoine	Nantes	France	CHU Hotel-Dieu
Jeanne	Mathieu	Lille	France	CHRU Lille
Rémerand	Francis	Tours	France	CHU Trousseau
Klouche	Kada	Montpellier	France	CHU Montpellier
Sztajnic	Simon	Toulouse	France	CHU Toulouse
Nouette	Karine	Bordeaux	France	CHU Bordeaux
Damien	Barraud	Metz	France	CH Metz Thionville
Rousseau	Anne-Françoise	Liège	Belgium	CHU Liège
Pantet	Olivier	Lausanne	Suisse	CHU Vaudois

## Appendix 2

### Postoperative infection criteria and definitions

Primary endpoint:

1. Sepsis and septic shock definitions [15]:
  - Sepsis is defined as life-threatening organ dysfunction caused by a dysregulated host response to infection.
  - Organ dysfunction can be identified as an acute change in total SOFA score ≥ 2 points consequent to the infection.
  - The baseline SOFA score can be assumed to be zero in patients not known to have preexisting organ dysfunction.
  - Septic shock is a subset of sepsis in which underlying circulatory and cellular/metabolic abnormalities are profound enough to substantially increase mortality.
  - Patients with septic shock can be identified with a clinical construct of sepsis with persisting hypotension requiring vasopressors to maintain MAP ≥ 65 mmHg and having a serum lactate level > 2 mmol/L (18 mg/dL) despite adequate volume resuscitation.
2. Surgical site infection criteria [12]:

Surgical site (operated skin) infection with general signs is considered a systemic infection originating from the skin. Treatment with systemic antibiotics for at least 5 days will be required to meet the endpoint.

The diagnosis of a skin infection is clinical.

1. *Positive local signs:*
  - Presence of a local or locoregional inflammatory reaction
  - Unfavorable and unexpected local evolution
  - Lysis of grafts
  - Necrosis of fat located under the graft

- To the operated healed areas
  - Impetigo
  - Lysis of healed areas

2 *Bacteriological skin samples:*

They are used to determine the germ(s) involved.

More often, a simple swab is enough. The biopsy is not systematic. It might be performed in difficult cases, followed by microbiology examination.

- Direct microscope examination with staining and semiquantitative measurement of germs
- Quantification of germs present per gram of tissue after homogenization: a threshold of 10^5^ CFU g^−1^ is retained as significant of the risk of hematogenous dissemination

An extemporaneous pathology examination after freezing enabling one to appreciate the level of invasion

- Colonization: germs in the nonvascularized tissue
- Infection: germs in the living tissue and in contact with vessels

3 Graft lysis needing a new graft procedure:

Graft lysis is defined as skin graft lysis needing a new skin graft, and this indication is assessed by a surgeon blinded to the randomization group within 7 days postsurgery.

## Appendix 3

### Steering committee

The steering committee is composed of the coordinating investigator François Dépret, the scientific director Matthieu Legrand, the statistician Pr Eric Vicaut, and investigators Pr Thomas Leclerc, Dr. François Ravat, Dr. Anne-Françoise Rousseau, Dr. Olivier Pantet, and Dr. Sandrine Wiramus.

## Abbreviations

BICU: Burn intensive care unit
TBSA: Total body surface area burn
eCRF: Electronic case report form
DSMC: Data and Safety Monitoring Committee
APHP: Assistance Publique Hôpitaux de Paris
DRCD: Clinical Research and Development Department
SAE: Serious adverse events
IRB: Institutional Review Board
