# Supplementary material for: The A2B trial, antibiotic prophylaxis for excision-graft surgery in burn patients: a multicenter randomized double-blind study
Source: Trials. 2020 Nov 25;21:973. doi: 10.1186/s13063-020-04894-y (PMC7687822; doi:10.1186/s13063-020-04894-y)
Supplement: Supplementary file 1 — Additional file 1. Details about quality control, case report form, management of noncompliance, audit and inspection, blinding methods, and serious adverse event notification [file 13063_2020_4894_MOESM1_ESM.docx]

**Supplementary material File 1**

**Quality control**

A Clinical Research Associate (CRA) appointed by the sponsor will be responsible for the proper conduct of the research, for collecting and documenting, recording and reporting the data generated in writing, in accordance with the Standard Operating Procedures applied within the DRCD and in accordance with French Good Clinical Practices as well as with the legislative and regulatory provisions in force.

The investigator and the members of the investigator's team agree to make themselves available during Quality Control visits carried out at regular intervals by the Clinical Research Associate. During these visits, the following elements will be reviewed:

- Written consent
- compliance with the research protocol and with the procedures defined therein
- Quality of the data collected in the case report form: accuracy, missing data, consistency of the data with the "source" documents (medical files, appointment books, original copies of laboratory results, etc.)
- Management of the treatments used
- Verification and transmission of occurred SAEs to the sponsor in accordance with the SAE grid.
- Verification of research product management through visits to the hospital pharmacy.
- For the closing Visit at each center: the Clinical Research Associate (CRA) will verify regulatory study documents maintained in the center and prepare consent envelopes for archiving.

**Case report form**

All information required according to the protocol must be entered in the case report forms. The case report forms has been validated by the coordinating investigator and the scientific director. The data must be collected as and when they are obtained, and clearly recorded in these case report forms. Each missing data item must be coded.

This digital case report form will be implemented in each of the centers owing to a web-based data collection tool. Investigators will be given a document offering guidance in using this tool.

When the investigators complete the case report via the Internet, the CRA can view the data quickly and remotely. The investigator is responsible for the accuracy, quality and relevance of all the entered data. In addition, the data are immediately verified as they are entered, owing to consistency checks. Thus, the investigator must validate any changes to the values in the case report form. These modifications will be subject to an audit trail. A justification can be added as a comment, when applicable. A print-out, authenticated (signed and dated) by the investigator, will be requested at the end of the research. The investigator must archive a copy of the authenticated document that was delivered to the sponsor.

Considering the type of study, the probability of missing patients is very low. In case of missing data for the main criterion an imputation by multiple imputation technic will be used.

**Management of non-compliances**

Any events that occur as a result the investigator or any other individual involved in conducting the study failing to comply with the protocol, standard operating procedures, good clinical practices or statutory and regulatory requirements must be recorded in a declaration of non-compliance and transmitted to the sponsor.

The sponsor has its own procedures for managing these non-compliances.

**Audits/Inspections**

The investigators agree to accept the quality assurance audits carried out by the sponsor as well as the inspections carried out by the competent authorities. All data, documents and reports may be subject to regulatory audits and inspections. These audits and inspections cannot be refused on the basis of medical confidentiality. An audit can be carried out at any time by independent appointed by the sponsor. The aim of the audits is to ensure the quality of the study, the validity of the results and compliance with the legislation and regulations in force. The persons who manage and monitor the trial agree to comply with the sponsor's requirements and with the competent authority regarding study audits or inspections.

The audit may encompass all stages of the study, from the development of the protocol to the publication of the results and the storage of the data used or produced as part of the study.

**Blinding methods**

The study will be kept blinded to patients, investigators and study personnel also during the entire study period.

The identification of treatment will be concealed by the use of a matching placebo to the study product that will be provided in boxes identical in packaging, labeling and appearance. In order to maintain the double blind, the preparation and the reconstitution of the treatments are carried out in the care service of each center by an independent manipulator of the follow-up and the evaluation of the patients. It guarantees blindness to the patient, the investigator and the rest of the health care team when preparing the treatment for the study. Opaques syringes will be used for administration to keep blindness between active drugs (opalescent) and the placebo (solvent).

**Serious adverse events notification**

According to Article R.1123-49 of the French Public Health Code (PHC), the investigator must notify the sponsor **without delay on the day when the investigator becomes aware** of any serious adverse event which occurs during a trial as described in Article L.1121-1(1) PHC, except those which are listed in the protocol (see section 10.1.2.2.2) and, if applicable, in the investigator's brochure as not requiring a notification without delay. These latter should be notified by the investigator to the sponsor in an appropriate delay taking into consideration the specific features of the trial, the serious adverse events and the modalities specified in the protocol or the investigator’s brochure.

The sponsor will especially follow the serious adverse events listed below:

- Events with fatal outcome
- Anaphylactic shock
- Quinck edema

The investigator must notify the sponsor without delay on the day when the investigator becomes aware of emerging safety issue, as well as security measures taken.

Other events require the investigator to notify the sponsor without delay:

- Liver tests abnormalities (ALT/AST three times higher than the upper limit)
- Clostridium-difficile colitis
- Acute kidney injury

All these events will be reported in the trial publication.

**Information of the person who volunteers to a research**

In accordance with Article L. 1122-1 of the Public Health Code, the person participating in the research received prior oral and written information on the research, allowing him or her to give free and informed consent. He or she is aware, in full and candid understandable terms, the objectives, risks and constraints of the research, the monitoring and security measures, the treatment of personal data necessary for the purpose of the research, the right to refuse to participate in the research or the possibility to withdraw consent at any time, etc... All above information must be included in a written document.

**Specificity of this study for obtaining consent of the person who is part of the research**

This research will be conducted in a context of inclusion under the emergency provisions of the law (Article L1122 -1-2 of the PHC / [Order n°2016-800 of June 16 2016 - art. 2](https://www.legifrance.gouv.fr/affichTexteArticle.do;jsessionid=90300EF9BD71990C38E69A63D62495ED.tpdila14v_3?cidTexte=JORFTEXT000032719520&idArticle=LEGIARTI000032720817&dateTexte=20170907&categorieLien=id#LEGIARTI000032720817)). Obviously, given the inclusion and non-inclusion criteria, the consent of patients or relatives if present cannot be always collected at baseline. Thus, given the extreme emergency situation not allowing collecting the prior consent of the person or relative, the protocol provides that their consent is not necessary. In case of positive outcome, a delayed consent will be asked to the patient for further participation to the study. The participant shall be informed regarding the study during his or her hospitalization as soon as his or her condition allows. Thereafter, if the patient agrees, he or she signs the delayed consent form to pursue his or her participation to the research and a “no objection to the use of his/her data" form for the possible continuation of this research will be completed.

According to the law (art. L1122-2 and L1121-8 of the Public Health Code), if the patient has a legal representative (guardian designated by law), it belongs to the patient's guardian to give his or her consent for a further participation to the research and the collection and the exploitation of the data.

**Informed consent documents**

The inform consent document includes an answer to the following questions:

-What is the purpose of this research?

- What does the research consist of?
- What is the timeline for the research?
- What are the benefits patient’s participation?
- What are authorized and unauthorized treatments?
- What are the foreseeable risks and constraints added by the research?
- What are the possible medical alternatives?
- What are the modalities of medical care at the end of the patient’s participation?
- How will the data collected for the research be processed?
- How is this research framed?
- What are the patient’s rights?
