# Supplementary material for: The A2B trial, antibiotic prophylaxis for excision-graft surgery in burn patients: a multicenter randomized double-blind study
Source: Trials. 2020 Nov 25;21:973. doi: 10.1186/s13063-020-04894-y (PMC7687822; doi:10.1186/s13063-020-04894-y)
Supplement: Supplementary file 3 — Additional file 3. Institutional review board (IRB) of Sud Est IV approval [file 13063_2020_4894_MOESM3_ESM.pdf]

President: Dr. Amandine BERTRAND REYNAUD
Vice-president: Marie-Amélie EUDELINE
Secretary general: Guillaume DUYCK

FAVOURABLE NOTICE

Interventional research category 1° L1121-1 of the Public Health Code MEDICAMENT(S)

**Identifiers**	**EudraCT: 2019-002396-34**	**CPP: 19.10.18.57044**	**ANSM: 2019-10-00036**
**Title**	***Antibioprophylaxis for excision-graft surgery in burn patient: a multicenter randomized double-blind study: A2B trial: A2B trial***		
**Coordinating investigator**	Dr François DEPRET, AP-HP Hôpital Saint Louis PARIS (75)		
**Promoter**	ASSISTANCE PUBLIQUE – HOPITAUX DE PARIS	Réf. Promoteur : APHP180605 / A2B trial	
**Applicant**	DRCI Carré historique Hôpital Saint-Louis 1, avenue Claude Vellefaux 75010 PARIS<br>Cécile KEDZIA / cecile.kedzia@aphp.fr		
**Reception date**	18/10/2019 and supplement the 30/10/2019	Answer after deliberation: 06/12/2019 and supplement the 11/12/2019	
**Documents**<br>*Complete list of annexed documents*	**Opinion request form (from the EudraCT application) of 11 December 2019**<br>**Protocol, version 1.1 of 4 December 2019**<br>**French Protocol Summary, version 1.1 of December 4, 2019 Summary of Product Characteristics :**<br>**PR1 : PIPERACILLIN TAZOBACTAM 4g/0.5 g of December 4, 2018 PR2 : CEFAZOLIN 2g of March 27, 2018**<br>**Information and Consent Documents, version 1.1 of December 4, 2019 (4): patient; patient suit; family/trusted person/parent; family/trusted person/parent suit**<br>**List of Investigators, Version 1.0 of September 23, 2019**		
**Session**	12 November 2019	Deliberation: A19-337	

Members present

TITULATORS	SUPPLIERS	TITULATORS	SUPPLIERS
**FIRST COLLEGE**		**SECOND COLLEGE**	
1. Research involving the human person:		1. Ethics: /	
Dr A.BERTRAND*<br>Dr P.CONY-MAKHOUL**<br>Mme R.MARAVAL-GAGET<br>Mme M.MONTANGE	Mme N.FALETTE**	2. Psychologist: Mme C.OLIVIER<br>3. Social worker: /<br>4. Legal	
		Mme M-A EUDELINE	Mme E.CHAPOUTIER
2. General practitioner:	Dr A.CERAULO*	5. Representatives of associations approved according to art. L1114-1 CSP :	
3. Hospital Pharmacist:	Mr M. PHILIPPE	Mme C.FABRY	
4. Nurse: Mr G.DUYCK			

* Pediatrician; ** Person qualified in biostatistics or epidemiology - Art. R.1123-11 CSP: "To be valid, the committee's deliberations require the presence of at least seven members, at least three of whom belong to the first college mentioned in Article R.1123-4, including at least one person qualified by virtue of his or her competence in biostatistics or epidemiology, including when they take part in the discussions by means of a conference call or audiovisual conference, and three belong to the second college comprising at least one representative of associations approved in accordance with the provisions of Article L. 1114-1. In accordance with Article L1123- 3, persons who are not independent of the sponsor and the research investigator do not take part in the deliberations concerned.

**Motivation**	The indication for antibiotic prophylaxis in burn patients remains highly controversial and has not yet reached consensus. The objective would be to reduce the risk of local or systemic post-operative infections. Surgery in burn patients is associated with a high risk of bacteremia, post-operative infections and sepsis. However, antibiotic prophylaxis exposes to the risk of selection of resistant pathogenic strains as well as adverse effects of antibiotics. The French Burn Society (SFB) and the French Society of Anaesthesia and Resuscitation (SFAR) suggest that antibiotic prophylaxis should be performed perioperatively but the lack of data prevents strong international recommendations on the best strategy to adopt. In addition, recommendations for perioperative prophylaxis vary among studies. Therefore, there is interest in determining whether perioperative systemic antibiotic prophylaxis could reduce the incidence of post-operative infections in burn patients. The main objective of the study is to evaluate the impact of systemic antibiotic prophylaxis in burn patients on post-operative infections, sepsis, graft lysis requiring a new skin graft within 7 days. Secondary objectives will seek to evaluate 90-day mortality, impact on antibiotic consumption, length of hospital stay and colonization by multi-resistant bacteria during the hospital stay.<br>This is a multi-centre, randomised, double-blind, phase 3 clinical trial. 506 patients aged 18 to 80 years, with a body surface area burned between 5 and 40% and requiring at least one excisional transplant surgery within 14 days, will be recruited by an investigator in the specialised services of the participating centres over an estimated period of 48 months. An emergency inclusion procedure in accordance with the provisions of Article L1122-1-3 of the Public Health Code is also provided for patients whose state of health justifies it. After information and consent, eligible patients will be randomized (1:1 ratio) between a group receiving antibiotic prophylaxis, depending on whether they are colonized (Piperacillin/Tazobactam 4g) or not (Cefazolin 2g) by pseudomonas aeruginosa, and a placebo group (0.9% NaCl solution). Treatments will be administered by IV 30 minutes before the procedure. Randomization is stratified according to the body surface area burned (5 to 20% and 21 to 40%). The same treatment will be administered if a second intervention is required. An evaluation is performed 7 days after intervention (clinical assessment, SOFA score). Patients will be followed up for 28 days (bacterial colonization) then 90 days (clinical examination, colonization, adverse events) after. A telephone collection is planned for patients discharged from hospital (vital status and adverse events).<br>The statistical analysis is described and the population size is justified. The evaluation criteria are adapted to the objectives. The computerised processing of personal data is declared by the promoter to be non-compliant with the reference methodology of the CNIL MR-01, and authorisation must be obtained prior to its implementation. This authorization must be specified in the information documents. The antibiotics being tested are known and widely used. The scientific rationale is relevant, particularly with regard to the use of a placebo. The information given to the patient and/or his/her representative (relative/trusted person/family) is clear. The balance of benefits and risks is acceptable.<br>Having regard to the public health code and in particular articles L. 1123-6 and L1123-7 and the regulatory provisions adopted for their application;<br>Having regard to the initial request for an opinion sent to the Committee; Having regard to the additional information provided by the promoter on 6 and 11 December 2019 at the request of the Committee in its deliberation of 12 November 2019,<br>**The Committee shall deliver a favourable opinion on the conditions for the validity of the research.**<br>If the research has not begun within two years, this opinion becomes null and void (Article R1123-26 of the Public Health Code).

Committee for the Protection of Individuals South-East IV

President: Dr. Amandine BERTRAND REYNAUD
Vice-president: Marie-Amélie EUDELINE
Secretary general: Guillaume DUYCK

<table>
<tr><td></td><td></td></tr>
<tr><td>CPP SOUTH-EAST IV
Centre Léon Bérard 28, rue Laennec - 69373 Lyon Cedex 08
Email: cppse4@lyon.unicancer.fr
Secretary: except on wednesday Tél.: 04 78 78 27 61 / Fax 04 78 78 28 58
Please quote the CPP reference of the file in all your correspondence.</td><td>Date and signature: 11th December 2019

President, Dr Amandine BERTRAND REYNAUD</td></tr>
</table>

Annex: Documents

ADMINISTRATIVE FILE	N° version	Date (jj/mm/aaaa)
- Clinical Trial Notice Request Letter<br>- Reply letter AR of 24/10/2019<br>- Authorization request letter (CAEC)		17/10/2019<br>28/10/2019<br>17/10/2019
Letter of response to deliberation A19-337		04/12/2019
Revised EudraCT Clinical Trial Notification Application Form (from EudraCT application)		11/12/2019
Additional document to the request for advice to the PPC		17/10/2019
**RESEARCH FILE**	**N° version**	**Date (jj/mm/aaaa)**
Clinical Trial Protocol* (signature page: sponsor (unsigned) and French Coordinator)	1.1	04/12/2019
Summary of the protocol in French*	1.1	04/12/2019
Summary of Product Characteristics (SPC) Experimental Drug(s) :<br>PR1: PIPERACILLIN TAZOBACTAM 4 g/0.5 g (piperacillin tazobactam Mylan 4 g/0.5 g, powder for solution<br>for infusion)<br>PR2: CEFAZOLIN 2g (Cefazolin Mylan 2 g, powder for injectable solution (IM-IV))		04/12/2018<br>27/03/2018<br>07/09/2018
PL1: SODIUM CHLORIDE 0.9% (sodium chloride 0.9% B. Braun, solution for injection in ampoule<br>Summary of the data justifying the use and safety of use of the investigational medicinal product(s) :<br>- Protocol (cf CAEC)		
- Antibioprophylaxis in surgery and interventional medicine (adult patients). Update 2010. Ann Fr Anesth Resuscitation. 2011 Feb ;30(2) :168-90<br>Backgrounder(s) *:<br>- Patient<br>- Patient pursuit after inclusion in an emergency situation	1.1<br>1.1<br>1.1<br>1.1	04/12/2019<br>04/12/2019<br>04/12/2019<br>04/12/2019
- Relative/trusted person/inclusive parent in emergency situations		
- Prosecution close to / trusted person / relative after inclusion in an emergency situation		02/07/2019
Consent form(s): included in the corresponding information documents		17/10/2019
Insurance certificate: HDI GLOBAL SE n°0100518814033 190089	1.0	23/09/2019
Justification adequacy of human, material and technical resources and compatibility with the safety requirements of the		2019

** Document(s) transmis en versions « suivi de modifications » (ou modifications surlignées)*
