# Supplementary material for: The A2B trial, antibiotic prophylaxis for excision-graft surgery in burn patients: a multicenter randomized double-blind study
Source: Trials. 2020 Nov 25;21:973. doi: 10.1186/s13063-020-04894-y (PMC7687822; doi:10.1186/s13063-020-04894-y)
Supplement: Supplementary file 4 — Additional file 4. Agence nationale de sécurité du medicament et des produits de santé approval [file 13063_2020_4894_MOESM4_ESM.pdf]

ansm
Agence nationale de sécurité du médicament
. et des produits de santé

CLINICAL TRIAL AUTHORIZATION OF MEDICINE FOR HUMAN USE

Date : 23 DEC. 2019

Clinical Trial Identifiers			
Title	Antibioprophylaxis for excision-graft surgery in burn patient: a multicenter randomized double-blind study: A2B trial		
Promoter	ASSISTANCE PUBLIQUE - HOPITAUX DE PARIS (APHP)		
Ref to remind	MEDAECNAT-2019-10-00036	N° EudraCT	2019-002396-34

Sender	Addressee (applicant: name / company / tel.)	
ANSM / INFHEP Product Management / Vaccines, antibiotics, antifungal and antiparasitic team	Cécile KEDZIA<br>DRCI Hôpital saint Louis<br>33 01 44 84 17 33	
File followed by: Perrine Nuez<br>Tél : 33 (0) 1 55 87 41 67 / Fax : 33 (0) 1 55 87 36 26<br>Mail : perrine.nuez@ansm.sante.fr	Mail	cecile.kedzia@aphp.fr

recipient CPP	South-East IV	Mail	cppse4@lyon.unicancer.fr

Having regard to the Public Health Code and in particular Article L. 1123-8, and the regulatory provisions adopted for its application, and having regard to the clinical trial authorisation application file sent to the National Agency for the Safety of Medicines and Health Products (ANSM) ,

Having regard to the additions made by the sponsor on 05/12/2019, 16/12/2019 and 19/12/2019 and in particular the protocol of the trial mentioned in the modified object (version 1.3 of 20/12/2019), following the request of the ANSM ;

The authorization referred to in Article L. 1123-8 of the Public Health Code is granted for the clinical trial.
cited in the subject line.

Î.3 thtf gF0#UÎtS
^'cins, antibiotiques, antifongiques, et antiparasitaires,
Direction des vaccins, des médicaments anti-infectieux
+n htpÛÎ0 gastro-entérologie, en dermatologie, de thérapie génique.
et des maladies métaboliques rares
Dr Isabelle PARENT DUCIJA ELET

I ask you to send any request for modifications concerning this file by email to the following address: ams-essaiscliniques@ansm.sante.fr. When sending these files, I ask you to make sure to write in the subject line of the message the mention: AMM/EURODRACT N° for MS submitted for authorization or for mixed files (including modifications submitted for authorization and others for information).

Confidentiality Confidentiality
This transmission is for the attention of the addressee(s) listed above only and may contain privileged and/or confidential information. If you are not the preferential or/and confidential infonnation. If you are not the inteiided recipient, you intended recipient or a person authorized to deliver this transmission, you are hereby notified that you have received the document by mistake and any use, disclosure, copying or communication of the content of this transmission is prohibited. content is prohibited. If you have received this transmission by mistake, please If you have received this transmission by inistake, please call us iminediately and inform us by phone immediately and return the original message by mail. return the original message by mail. Thank you.
Thank you. code: AEC_E OR004 v03
